# Disulfiram activates autophagy via proteasome inhibition and c-Fos/beclin-1 upregulation, synergizing with chloroquine

**DOI:** 10.1038/s41420-025-02899-7

**Published:** 2025-12-12

**Authors:** Kejin Wang, Zhen Wang, Wenxuan Peng, Gen Li, Honglin Xiao, Ziqi Zhong, Yilin He, Yingnan Yu, Yijiang Song, Li Xiang, Changjie Wu

**Affiliations:** 1 Guangdong Provincial Key Laboratory of Gastroenterology, Department of Gastroenterology, Nanfang Hospital, Southern Medical University, Guangzhou, China; 2 Department of Laboratory Medicine, Guangdong Provincial Key Laboratory of Precision Medical Diagnostics, Guangdong Engineering and Technology Research Center for Rapid Diagnostic Biosensors, Guangdong Provincial Key Laboratory of Single-cell and Extracellular Vesicles, Nanfang Hospital, Southern Medical University, Guangzhou, China; 3 Department of Gastroenterology, The Second Affiliated Hospital, School of Medicine, The Chinese University of Hong Kong, Shenzhen & Longgang District People’s Hospital of Shenzhen, Shenzhen, China

**Keywords:** Chemotherapy, Colorectal cancer

## Abstract

Disulfiram (DSF), a clinically approved anti-alcoholism drug, exerts anti-tumor activity through its copper metabolite CuET by inhibiting the ubiquitin-proteasome system (UPS). However, its regulatory mechanisms on autophagy and potential for combination therapy remain to be clarified. Here, we revealed that DSF activates autophagy in colorectal cancer (CRC) cells via dual mechanisms: compensatory autophagy induction through proteasome inhibition by targeting the p97-NPL4 axis, and transcriptional upregulation of the autophagy-related gene *BECN1* via *FOS* gene activation. Transcriptomic analysis identified that DSF enhances c-Fos expression, promoting c-Fos/AP-1 complex binding to the *BECN1* promoter to drive beclin-1 expression. Furthermore, combining DSF with the autophagy inhibitor chloroquine (CQ) synergistically enhanced anti-tumor efficacy both in vitro and in vivo. DSF-induced autophagy may mitigate its pro-apoptotic effects, while autophagy inhibition fully blocks protein degradation pathways, leading to lethal protein accumulation. This study elucidates DSF’s dual regulation of autophagy through UPS suppression and the c-Fos/beclin-1 axis, and validates the synergistic efficacy of DSF combination with CQ in CRC, providing a theoretical foundation and translational potential for DSF-based combination therapies.

## Introduction

Cancer is one of the leading causes of death worldwide [1]. Despite advancements in surgery, radiotherapy, and immunotherapy, chemotherapy remains one of the most effective cancer treatments [2]. Therefore, developing novel anticancer drugs is of great importance. However, the development of new anticancer drugs is often time-consuming and expensive. To overcome these challenges, drug repurposing has emerged as an alternative strategy. By identifying new indications for already approved drugs with well-established toxicological and pharmacokinetic profiles [3, 4], this approach can significantly reduce costs and shorten the timeline for discovering new treatment options [5].

Disulfiram (DSF), as an anti-alcoholic drug, has been in clinical use for over 70 years [6]. Its efficacy in alcohol aversion relies on inhibiting aldehyde dehydrogenase (ALDH), leading to the accumulation of acetaldehyde and causing an adverse reaction to alcohol consumption. In recent years, growing evidence has demonstrated that beyond its use in anti-alcoholism, DSF exhibits antitumor activity against a variety of cancers, including breast cancer, prostate cancer, and glioblastoma [7–9]. While the mechanisms of its antitumor effects remain not fully clarified, recent studies suggest that DSF’s anticancer activity is largely mediated by its copper-containing metabolite, CuET, rather than ALDH inhibition [10]. CuET binds NPL4 and induces its aggregation, consequently disabling the vital p97–NPL4–UFD1 pathway, leading to the accumulation of polyubiquitinated proteins and ultimately inducing cell death [11]. Despite its promising efficacy, clinical trials using DSF or in combination with other anticancer drugs have not yielded satisfactory therapeutic outcomes, highlighting the need for new combinatorial strategies [12–14].

Autophagy is a critical intracellular degradation process that allows cells to eliminate damaged organelles, misfolded proteins, and other cellular debris, thereby maintaining homeostasis [15, 16]. It plays a dual role in cancer treatment. On one hand, autophagy enables cancer cells to survive under stress caused by chemotherapy, radiation, or targeted therapies [17–19]. On the other hand, inhibition of autophagy has been shown to enhance the efficacy of these treatments [20–22]. Thus, combining autophagy inhibitors with chemotherapy has emerged as a promising clinical strategy and an important direction for new drug development.

Here, we found that DSF treatment enhances autophagy through two key pathways, inhibiting the ubiquitin-proteasome system (UPS) and promoting c-Fos/beclin-1 expression. Considering that intracellular protein degradation is mainly governed by the UPS and the autophagy-lysosome pathway, DSF inhibits the ubiquitin-proteasome pathway while promoting autophagy, leading to a cellular dependence on autophagic degradation. Therefore, autophagy inhibitors can significantly enhance the anticancer efficacy of DSF. Therefore, we provided that an autophagy inhibitor combined with DSF could be a novel and translational combination therapy strategy for cancer treatment.

## Results

### DSF induces autophagy in CRC cells

**Fig. 1:**
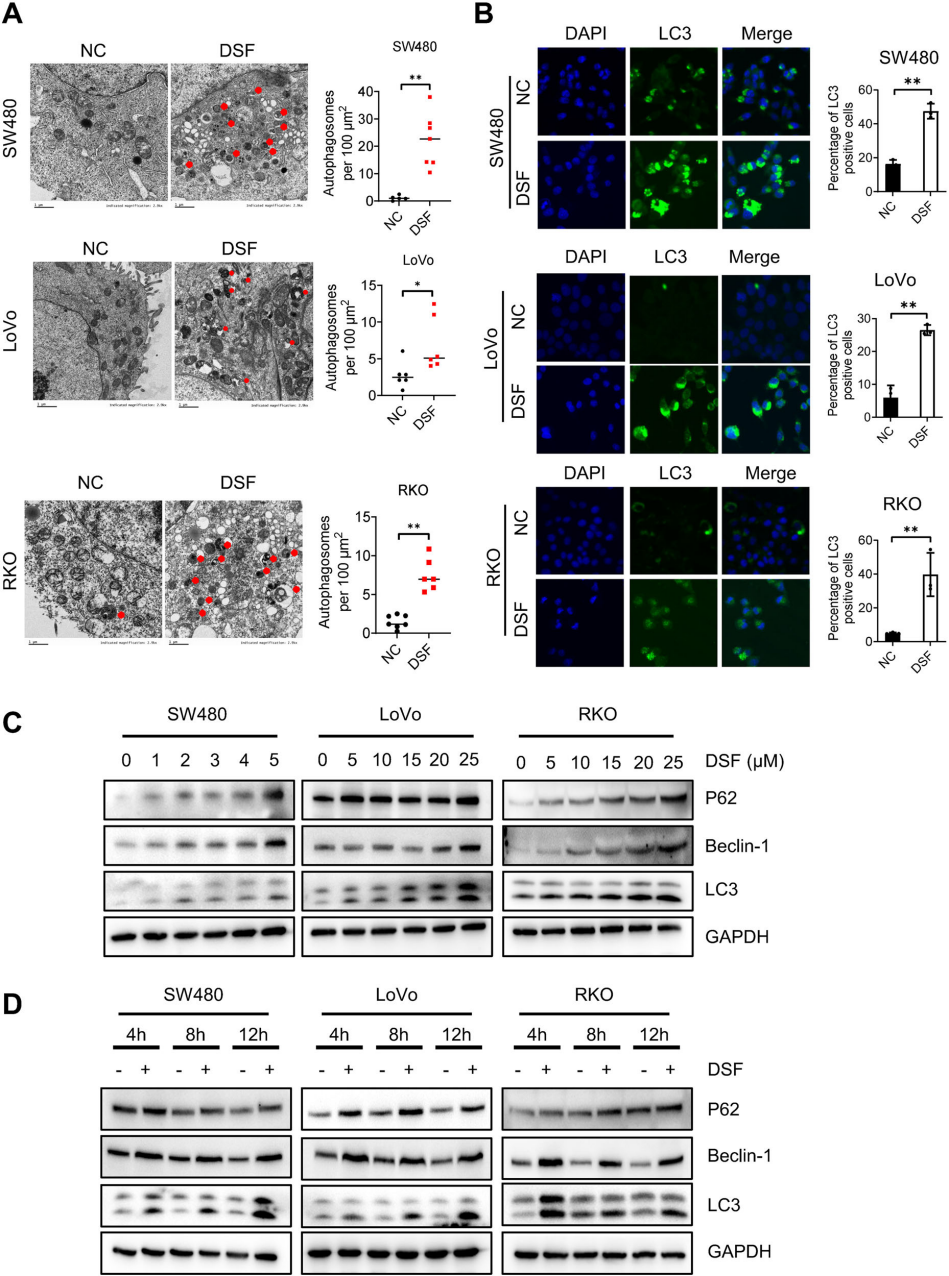
DSF induces autophagy in CRC cells. **A** TEM image of autophagosomes and lysosomes in DSF-treated SW480, LoVo, and RKO cells, with a bar graph on the right showing the total number of autophagosomes and lysosomes per 100 μm² field of view. **B** Confocal microscopy image of LC3 immunofluorescence staining. **C** Western blot analysis of autophagy-related protein expression in SW480, LoVo, and RKO cells treated with increasing concentrations of DSF. **D** Western blot analysis of autophagy-related protein expression in SW480, LoVo, and RKO cells at different time points following DSF treatment. Data are presented as mean ± SD (*p < 0.05, **p < 0.01).

To test the effect of DSF on autophagy, we first employed transmission electron microscopy (TEM) to analyze the cytoplasmic changes in cells treated with DSF [23]. The results demonstrated a significant increase in the number of autophagosomes and lysosomes within the cytoplasm following DSF treatment (Fig. 1A). Immunofluorescence staining further confirmed a marked upregulation of LC3 levels in these cells post-DSF treatment (Fig. 1B). Subsequently, we examined the expression levels of autophagy-related proteins in SW480, LoVo, and RKO cells treated with DSF at varying time points or concentrations. Our findings revealed a dose- and time-dependent upregulation of LC3 protein expression in these cell lines (Fig. 1C, D). Collectively, these data indicate that DSF significantly enhances autophagy levels in colorectal cancer (CRC) cells.

### DSF-induced autophagy is mediated by proteasome inhibition via NPL4 in CRC cells

**Fig. 2:**
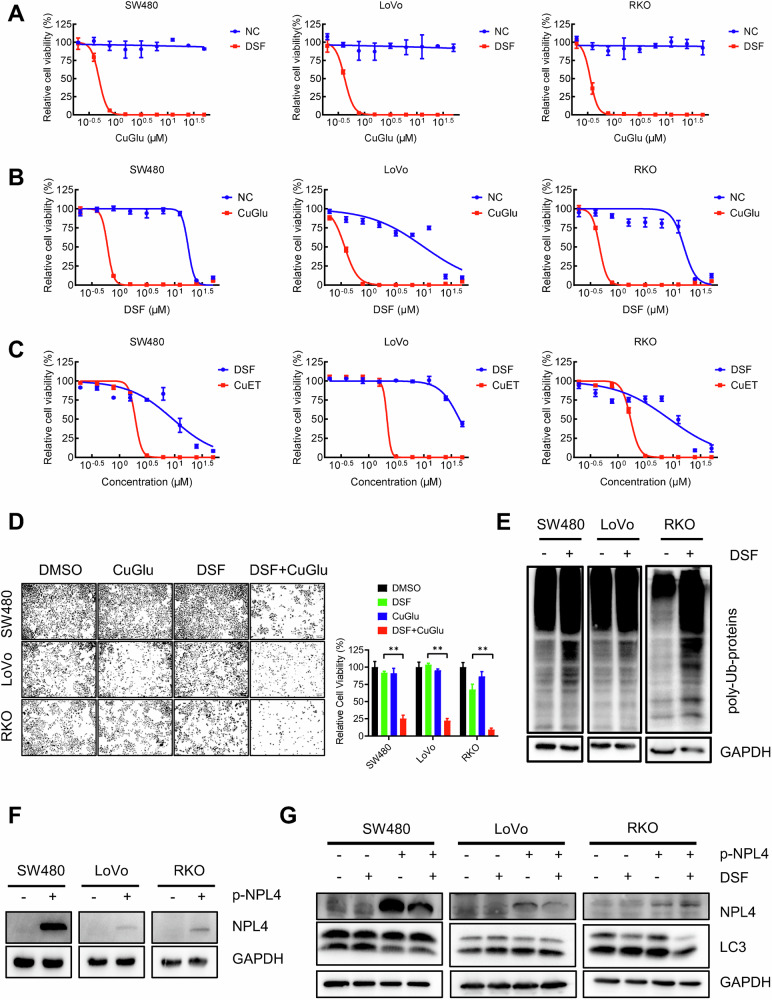
DSF-induced autophagy is mediated by NPL4. **A** CuGlu dose response curve with or without DSF in SW480, LoVo, and RKO cells. **B** DSF dose response curve with or without CuGlu in SW480, LoVo, and RKO cells. **C** Dose response curve of DSF and CuET in SW480, LoVo, and RKO cells. **D** Cell viability treated with DMSO, CuGlu, DSF, and DSF + CuGlu. **E** Western blotting detection of ubiquitination levels in SW480, LoVo, and RKO cells (**F**) verification of NPL4 overexpression. **G** Autophagy levels in SW480, LoVo, and RKO control cells and NPL4 overexpressing cells after DSF treatment. Data are presented as mean ± SD (**p < 0.01).

Protein degradation pathways are primarily categorized into two major systems: the UPS and autophagy. Previous studies have suggested that the antitumor activity of DSF largely depends on its copper-containing metabolite CuET, which induces cell death by inhibiting the ubiquitin-proteasome system and thereby blocking protein degradation [11]. Based on this, we hypothesized that the inhibition of the proteasome by DSF may lead to the promotion of compensatory autophagy. To test this hypothesis, we first validated the copper dependence of DSF. While copper gluconate (CuGlu) alone had no effect on tumor cells, its combination with DSF significantly reduced tumor cell viability (Fig. 2A). Compared with the control group, CuGlu markedly enhanced the antitumor activity of DSF (Fig. 2B), indicating that the effect of DSF is largely dependent on copper ions. Furthermore, CuET exhibited substantially stronger cytotoxicity than DSF (Fig. 2C). Consistently, in SW480, LoVo, and RKO cells, DSF combined with CuGlu significantly enhanced tumor cell killing at equivalent concentrations (Fig. 2D). We next evaluated the level of ubiquitination following DSF treatment in SW480, LoVo, and RKO cells. The results revealed a significant increase in ubiquitination levels, indicating effective inhibition of the proteasome (Fig. 2E). To test whether DSF-induced autophagy is NPL4 dependent, we overexpressed NPL4 in all three tested CRC cell lines (Fig. 2F). The results showed that NPL4 overexpression attenuated DSF-induced autophagy upregulation, suggesting that DSF-induced autophagy can be affected by DSF-induced proteasome inhibition (Fig. 2G).

### DSF promotes autophagy via activation of the c-Fos/beclin-1 axis in CRC cells

**Fig. 3:**
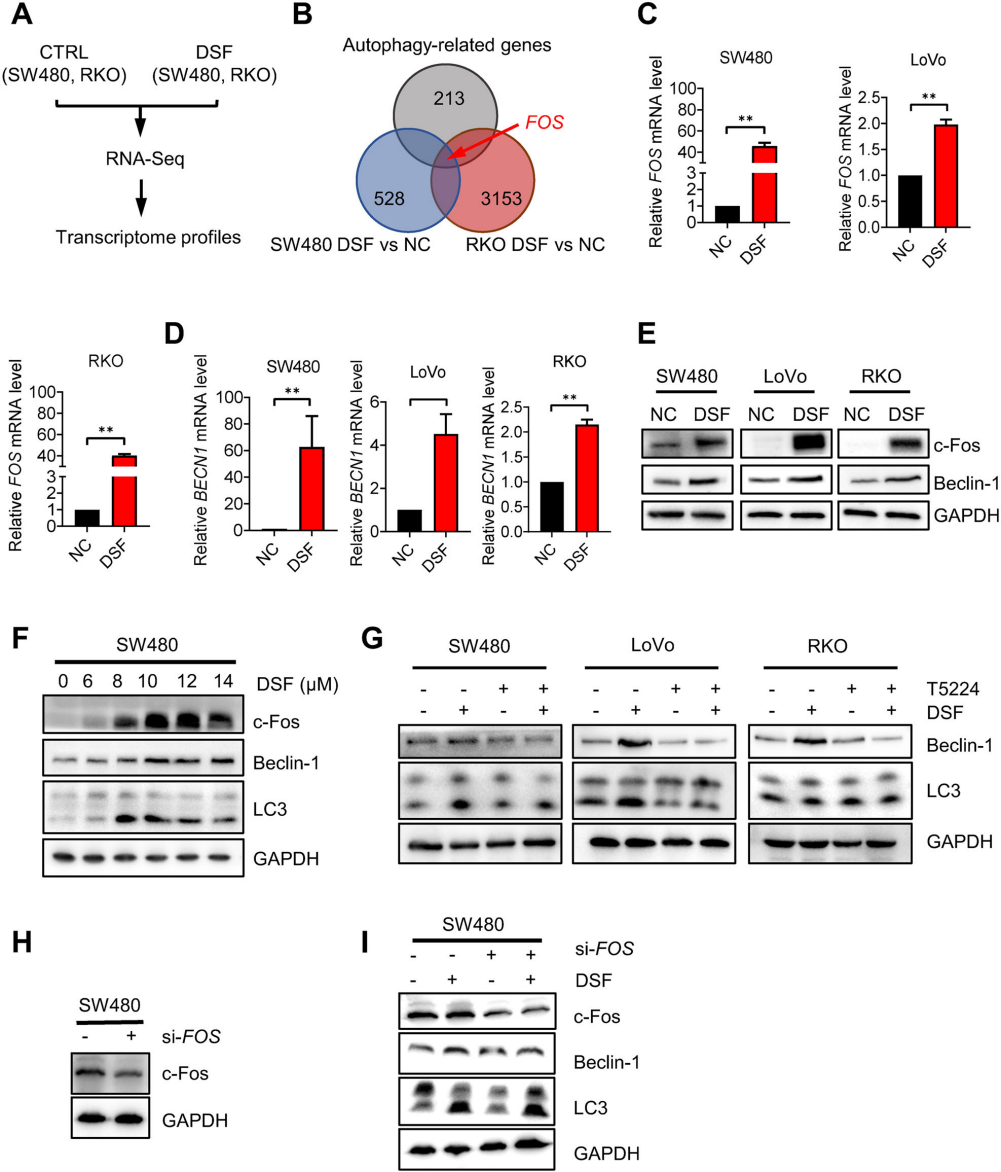
DSF-induced autophagy is mediated via the c-Fos/beclin-1 axis. **A** Design of experimental groups for transcriptome sequencing analysis. **B** Venn diagram summarizing the overlap between autophagy-related genes and differentially expressed genes from RNA-Seq (SW480, RKO). **C** Relative RNA levels of FOS in SW480, LoVo, and RKO cells as detected by qPCR after DSF treatment. **D** Relative RNA levels of beclin-1 in SW480, LoVo, and RKO cells as detected by qPCR after DSF treatment. **E** Protein expression levels in SW480, LoVo, and RKO cells following DSF treatment. **F** Protein expression levels in SW480 cells treated with varying concentrations of DSF. **G** Protein expression levels in SW480, LoVo, and RKO cells co-cultured with DSF and T5224. **H** *FOS* silencing efficiency detection by Western blotting. **I** Cells were pretreated with si-*FOS* and then treated with DSF. The expression levels of c-Fos, beclin-1, and LC3 were detected by Western blotting. Data are presented as mean ± SD (**p < 0.01).

Given that DSF can regulate gene transcription through epigenetic mechanisms, we next investigated whether DSF modulates autophagy at the transcriptional level. We first conducted transcriptomic analysis of DSF-treated SW480 and RKO cells using RNA sequencing and compared their mRNA expression profiles (Fig. 3A). The results showed that the *FOS* gene is the only autophagy-related gene which was significantly upregulated in both SW480 and RKO cells following DSF treatment (Fig. 3B and Supplementary Fig. 2). Notably, c-Fos, encoded by the *FOS* gene and acting as a core component of the AP-1 transcription factor complex, has been reported to directly bind to the promoter region of the *BECN1* gene (-582 to -574 bp TGAGTCA site), thereby activating the expression of the autophagy core regulator beclin-1 through dopamine D2/D3 receptor agonists such as quinpirole [24]. We postulated that DSF upregulates the expression of the *FOS* gene, thereby activating this pathway and inducing autophagy. Real-time fluorescence quantitative PCR (qPCR) confirmed that the transcription levels of c-Fos and beclin-1 were significantly increased after DSF treatment (Fig. 3C, D). Western blotting experiments further corroborated these findings (Fig. 3E), with DSF showing a dose-dependent upregulation of c-Fos protein levels (Fig. 3F). Moreover, treatment with the c-Fos/AP-1 inhibitor T-5224 diminished the DSF-induced autophagy upregulation in SW480, LoVo, and RKO cells (Fig. 3G). Upon silencing *FOS* using small interfering RNA, a similar attenuation in the upregulation of autophagy was observed (Fig.3H, I). These results demonstrate that DSF promotes autophagy by activating *FOS* transcription and promoting c-Fos/beclin-1 axis activity.

### Chloroquine (CQ) enhances the antitumor effect of DSF in CRC cells

**Fig. 4:**
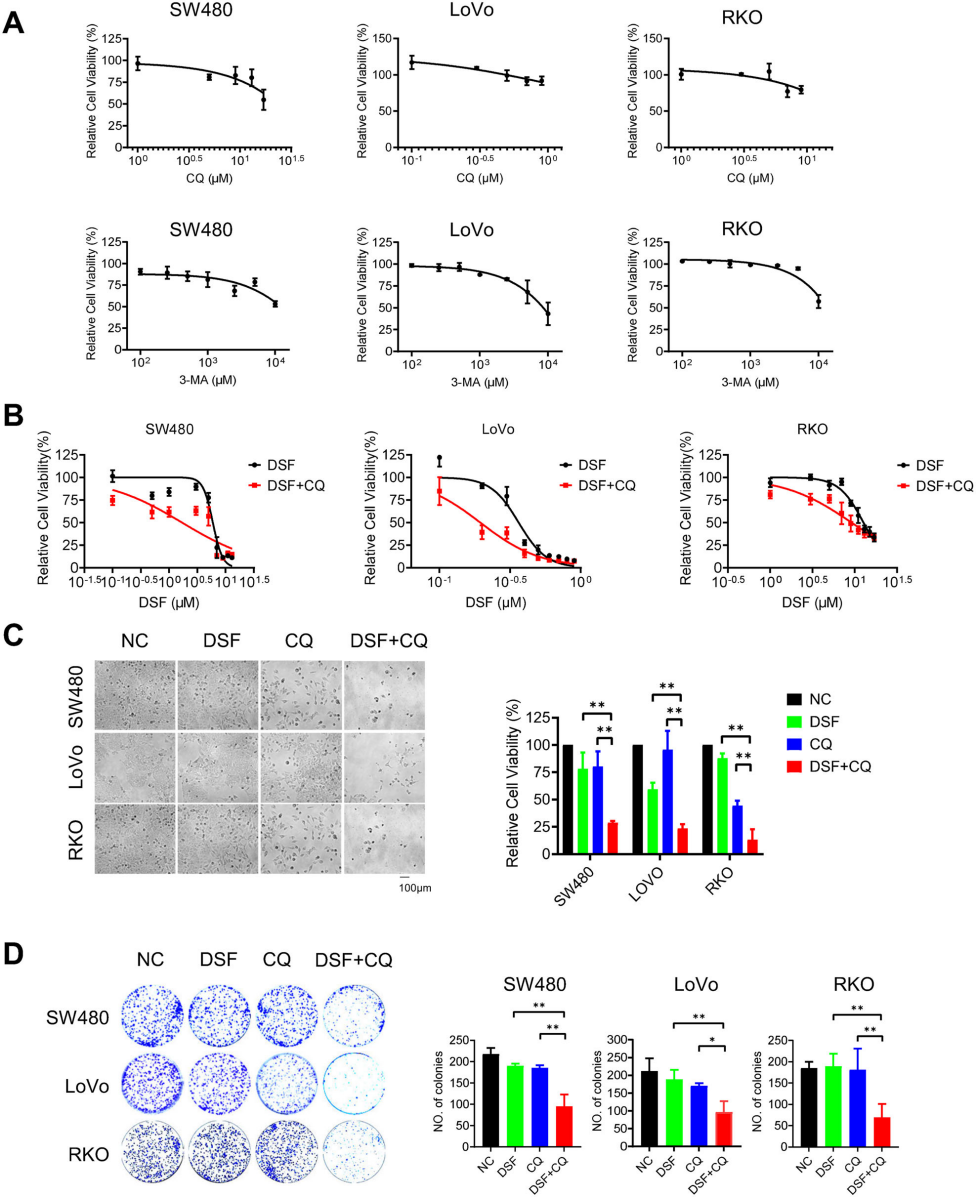
CQ enhances the antitumor effect of DSF. **A** CQ and 3-MA dose-effect curves of SW480, LoVo, and RKO. **B** DSF and CQ dose-effect curves of SW480, LoVo, and RKO. **C** Cell viability assay after drug treatment. **D** Colony formation assay after drug treatment. The images are the clone formation of SW480, LoVo, and RKO after staining, and the bar charts are the clone formation numbers calculated by ImageJ software. The experiment was repeated three times. Data are presented as mean ± SD (*p < 0.05, **p < 0.01).

DSF can inhibit the ubiquitin-proteasome system and induce cell death, which may be alleviated through the promotion of autophagy. Therefore, we hypothesized that combining DSF with autophagy inhibitors might achieve a stronger therapeutic effect. To test this hypothesis, we first evaluated the anti-tumor activity of the autophagy inhibitors CQ and 3-MA in three CRC cell lines (SW480, LoVo, and RKO). Since CQ is more widely used in clinical practice and our experimental results showed that its anti-cancer activity was superior to that of 3-MA, we selected CQ for subsequent studies (Fig. 4A). To further explore the sensitizing effect of CQ on DSF, we tested the impact of different concentrations of CQ on the survival rate of SW480, LoVo and RKO cells at a fixed DSF concentration. The results showed that as the concentration of CQ gradually increased, the combined treatment of DSF and CQ exhibited significantly enhanced cytotoxicity against CRC cells, outperforming the use of either drug alone (Fig. 4B). Additionally, under the condition of a fixed CQ concentration and varying DSF concentrations, the aforementioned synergistic effect was also significant. Subsequently, we further verified the inhibitory effect of the combined treatment of DSF and CQ on cell proliferation, finding that the inhibitory effect was significantly higher than that of the single drug groups. Meanwhile, the results of the colony formation assay indicated that the number of colonies formed in the DSF and CQ combination group was significantly lower than that in the other groups. These results suggest that CQ can effectively enhance the sensitivity of cells to DSF (Fig. 4C, D).

### Combination of DSF and CQ enhances cell apoptosis in CRC cells

**Fig. 5:**
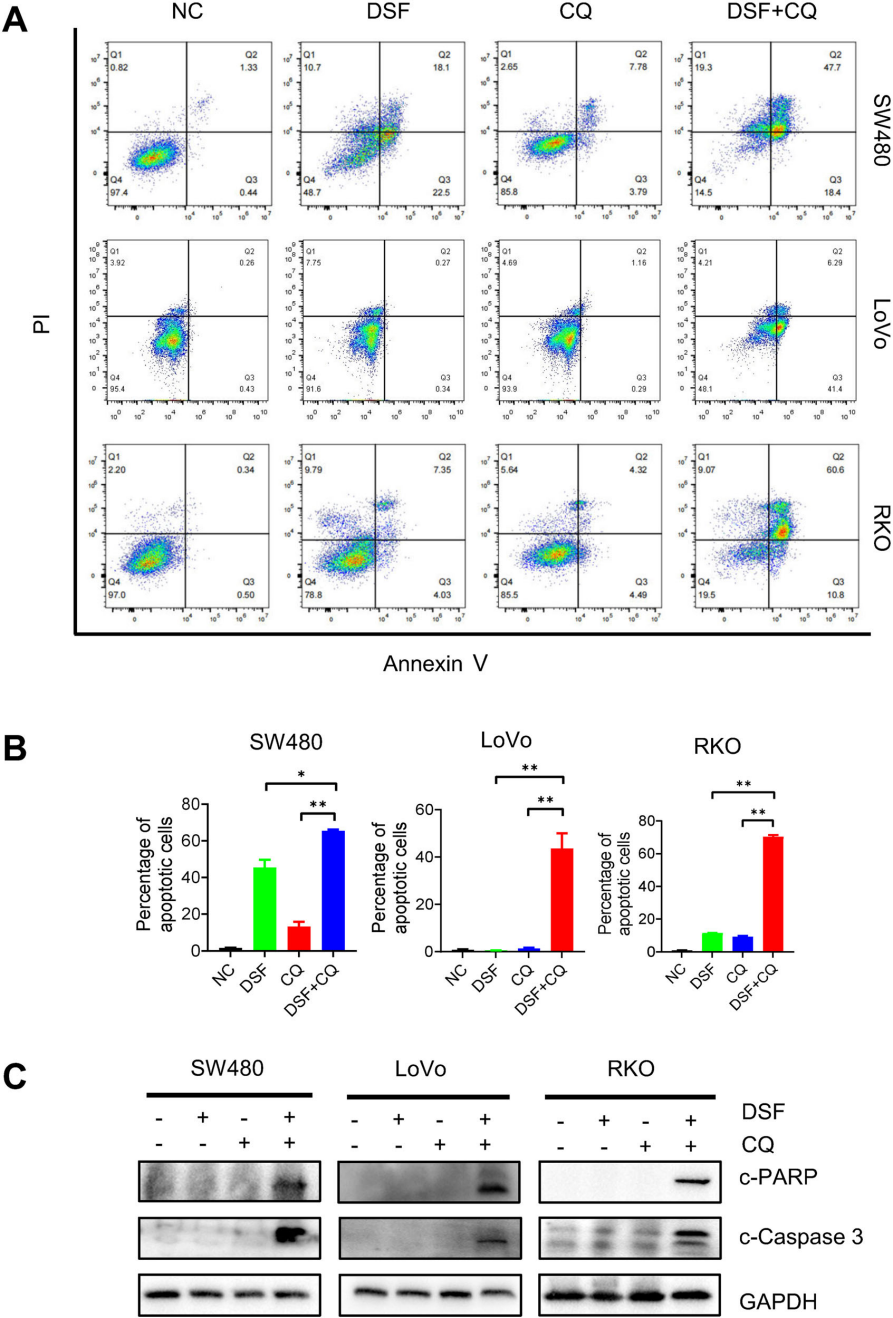
The combination of DSF and CQ enhances apoptosis. **A**, **B** Flow cytometry plots after treatment with DSF and CQ drugs. **C** Western blotting of proteins. Detection of apoptosis proteins PARP and Caspase-3 in SW480, LoVo, and RKO. Data are presented as mean ± SD (*p < 0.05, **p < 0.01).

To further investigate the cytotoxic effect of DSF and CQ on CRC, we employed flow cytometry and Western blot (WB) to assess apoptosis levels in SW480, LoVo, and RKO cells treated with the two drugs. The results demonstrated an increase in apoptotic cells and upregulated expression of apoptotic markers PARP and Caspase-3, indicating that the combination of DSF and CQ is more likely to induce apoptosis (Fig. 5A–C).

### DSF and CQ combination exhibits synergistic antitumor efficacy in vivo

**Fig. 6:**
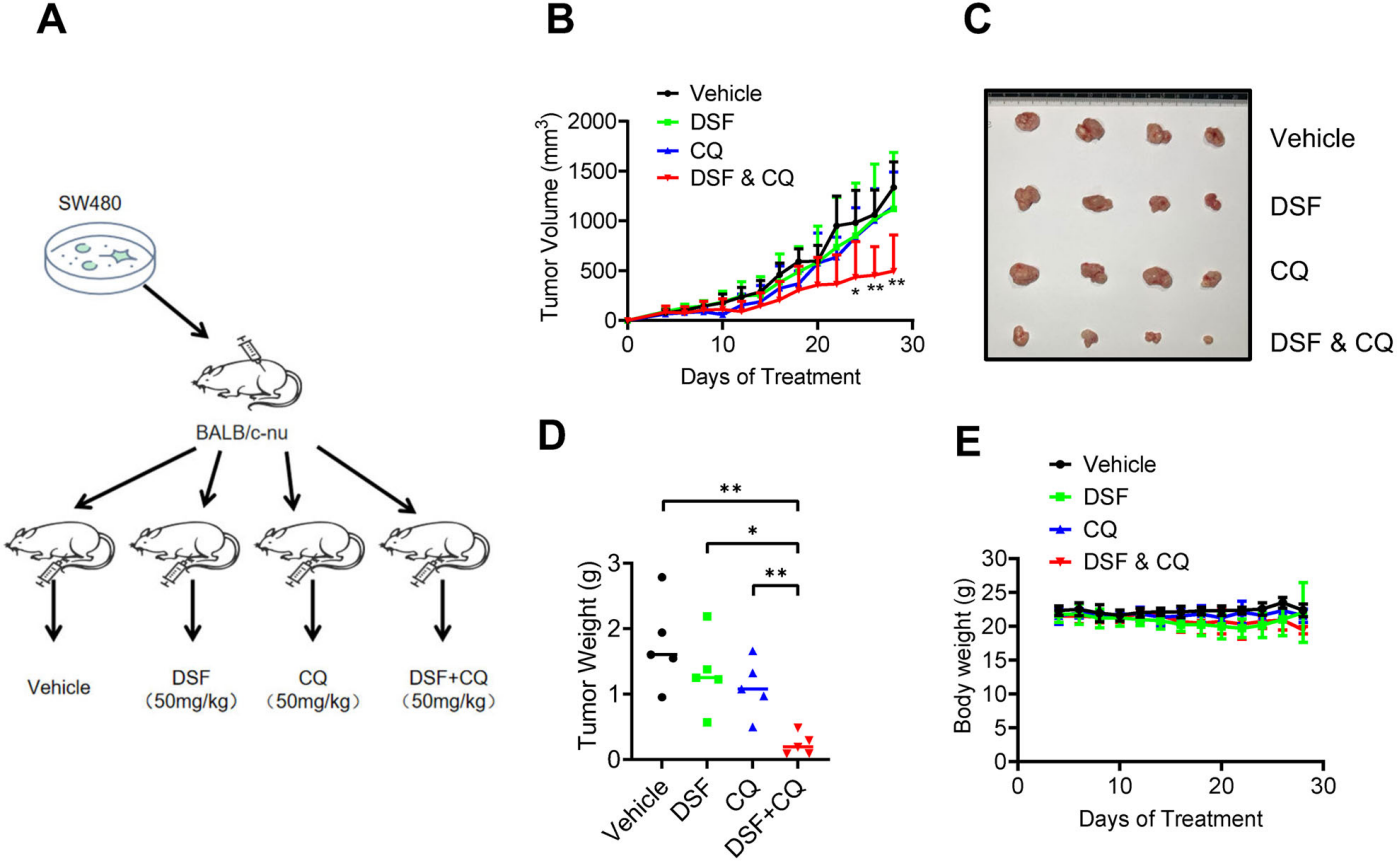
DSF and CQ synergistically enhance antitumor efficacy in vivo. **A** Schematic diagram of the mouse experiment. **B** Tumor growth curve in nude mice after injection of vehicle, DSF, CQ, and DSF + CQ. **C** Representative images of tumor size treated with vehicle, DSF, CQ, and DSF + CQ. **D** Wet weight measurement of the tumors isolated from mice. **E** Body weight of mice treated with vehicle, DSF, CQ, and DSF + CQ. Data are presented as mean ± SD (*p < 0.05, **p < 0.01).

To evaluate the in vivo anti-tumor efficacy of DSF in combination with CQ for CRC treatment, a subcutaneous xenograft tumor model was established using SW480 cells in this study (Fig. 6A). Following a 28-day intervention with drug administration every two days, tumor growth curve analysis revealed that the DSF combination with CQ demonstrated significantly greater growth inhibition compared to the single-agent groups and the control group (Fig. 6B). On the final experimental day, the tumor volume and weight in the combination treatment group were markedly reduced compared to those in the control and single-agent groups (Fig. 6C, D). Additionally, dynamic body weight monitoring data indicated minimal fluctuations in body weight during the combination treatment, with no significant weight loss observed, suggesting favorable safety of this regimen (Fig. 6E). Collectively, these findings demonstrate that the DSF and CQ combination regimen exhibits synergistic anti-tumor effects and good safety in the subcutaneous xenograft tumor model of CRC, highlighting its potential clinical translational value.

**Fig. 7:**
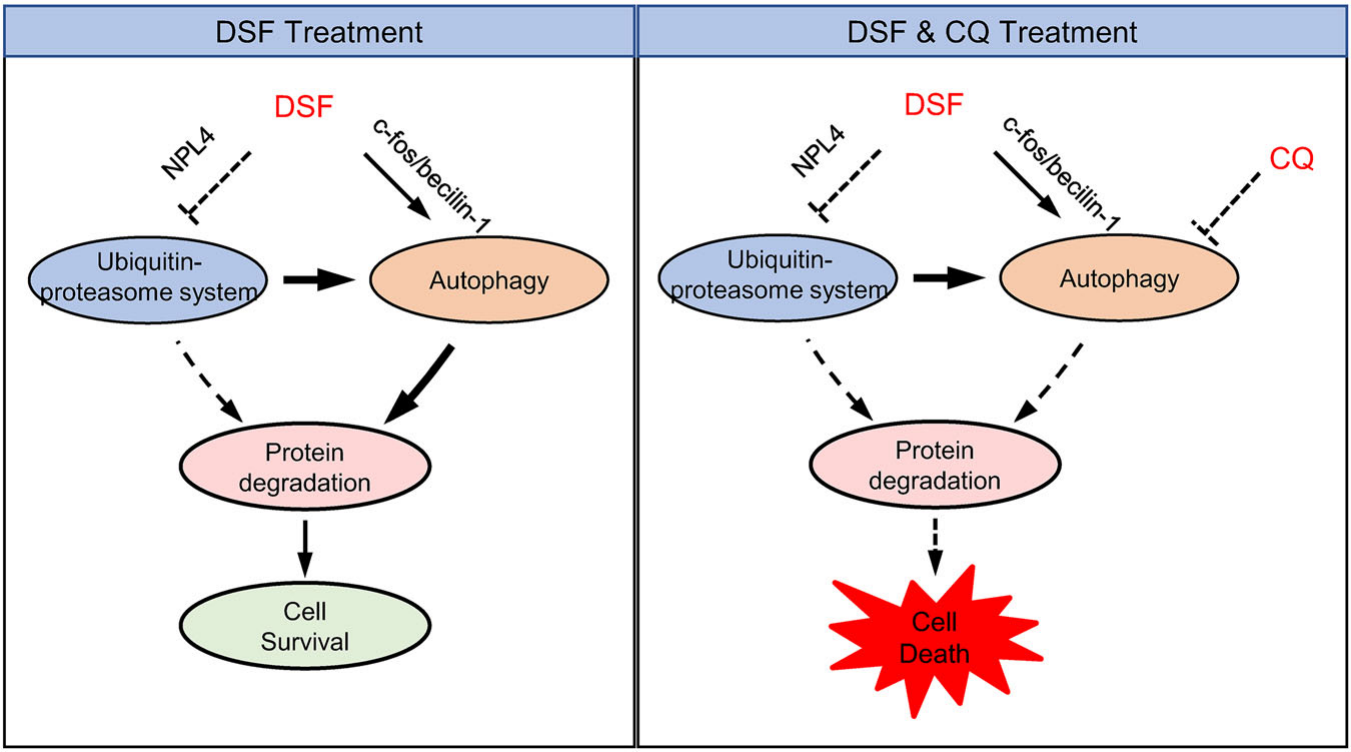
The working model of DSF combined with autophagy inhibitors in the treatment of CRC.

In summary, here we found that DSF promotes autophagy through two pathways to reduce its induced cell death, compensatory enhancement and activation of the c-Fos/beclin-1 axis (Fig. 7). The combination of DSF with autophagy inhibitors enhances cell death both in vitro and in vivo, thereby improving the anti-tumor efficacy of DSF. This strategy provides a novel therapeutic approach for combination cancer therapy.

## Discussion

DSF, as an FDA-approved anti-alcoholism drug, has recently demonstrated anti-tumor activity, which is mediated by its copper-containing metabolite CuET [25–29]. This activity depends on the inhibition of the p97-NPL4-UFD1 signaling pathway, thereby suppressing the ubiquitin-proteasome degradation system, and this activity is independent of its ALDH-inhibitory properties. Our study reveals that DSF promotes autophagy in tumor cells through two primary mechanisms: (1) compensatory enhancement of autophagy due to proteasome inhibition, as evidenced by partial suppression of autophagy upon NPL4 overexpression; and (2) transcriptional upregulation of the autophagy-related gene beclin-1 via enhanced expression of the transcription factor c-Fos. These findings establish novel mechanisms underlying DSF-induced autophagy.

Cellular protein degradation primarily occurs through two pathways: the UPS and autophagy [30]. While DSF inhibits the ubiquitin-proteasome pathway by targeting the p97-NPL4-UFD1 axis [11], it paradoxically activates autophagy, which may attenuate its anti-tumor efficacy [31–34]. Combining DSF with autophagy inhibitors completely blocks cellular protein degradation, leading to abnormal protein accumulation and subsequent cell death. Similar dual-target inhibition strategies have been documented in recent studies [35]. This synergistic approach significantly enhances DSF’s anti-tumor activity and therapeutic efficacy, suggesting a promising strategy for combination cancer therapy.

The anti-tumor activity of DSF is primarily attributed to CuET, its copper-complexed metabolite, which exhibits tumor tissue-specific accumulation. The CuET levels in tumor tissues were nearly one order of magnitude higher than those in non-tumor tissues [11]. Given the clinical availability of autophagy inhibitors such as CQ and the tumor-specific targeting of CuET, the combination of DSF and CQ may enable selective tumor cell killing while sparing normal tissues [36].

In summary, this study elucidates novel mechanisms of DSF-induced autophagy and demonstrates that combining autophagy inhibitors with DSF enhances its anti-tumor efficacy in CRC models. These findings provide a rationale for developing DSF-based combination therapies, offering a potential advancement in cancer treatment strategies.

## Materials and methods

### Cell culture and reagents

Human CRC cell lines SW480, LoVo, and RKO cells were obtained from the American Type Culture Collection (ATCC, Manassas, VA, USA). All cells were cultured in Gibco Modified Dulbecco’s Eagle Medium (DMEM) supplemented with 10% FBS and 1% penicillin/streptomycin at 37 °C in a humidified 5% CO_2_ atmosphere. DSF (T0054), T5224 (T5416), and CQ (T8689) were purchased from TargetMol (Waltham, MA, USA).

### Transfection

For the transfection of pCDH-CMV-NPLOC4 (provided by HanYi Biotech, Guangzhou) and si-FOS (synthesized by Tsingke Biotech, Beijing), Lipofectamine 3000 (Invitrogen, USA) was utilized to prepare the transfection reagent and conduct transfection in SW480, LoVo, and RKO cells. Following transfection, the cells were incubated for 48 h. The siRNA sequences are as follows: 5’-GCAUGGAGCUGAAGACCGA-3’.

### Immunoblot and antibodies

Whole-cell protein extracts were prepared using ice-cold RIPA buffer, composed of 25 mM Tris-HCl (pH 7.6), 150 mM NaCl, 1% NP-40, 1% sodium deoxycholate, and 0.1% sodium dodecyl sulfate (SDS). The protein sample was separated by SDS-polyacrylamide gel electrophoresis (SDS-PAGE) and subsequently transferred onto a nitrocellulose membrane. The membranes were blocked in 5% BSA for 1 h and then incubated with primary antibodies at 4 °C overnight. The primary antibodies for immunoblot analysis were listed as follows: LC3A/B (CST, #12741), SQSTM1/p62 (CST, #5114), beclin-1 (CST, #3495), c-Fos (Proteintech, #66590-1-Ig), Cleaved-caspase3 (CST, #9661), Cleaved-PARP (CST, #5625), Ubiquitin(A-5) (Santa Cruz Biotechnology, sc-166553), GAPDH (CST, #2118). Horseradish peroxidase-conjugated secondary antibodies were labeled with HRP (ZEN-Bioscience, #511203). Key WB results were quantified using Image J software, and the corresponding data are presented in Supplementary Fig. 1. Uncropped versions of all blots are shown in Supplementary Figs. 3 and 4.

### Tumor xenograft mouse model

CRC cells (SW480, 5 × 10^6^) were subcutaneously inoculated into 6-week-old female BALB/c nude mice. Upon tumor palpation, the mice were randomly divided into four groups, with five mice in each group (*n* = 5): (1) control group; (2) DSF (50 mg/kg) group; (3) CQ (50 mg/kg) group; and (4) DSF and CQ group. The drugs were dissolved in the vehicle (sterile saline containing 5% dimethyl sulfoxide, 5% Tween-80, and 30% polyethylene glycol-400). Tumor volumes were measured with a vernier caliper every two days for 28 days and calculated using the modified ellipsoid formula (long axis × short axis × short axis × 0.5). At the end of the experiment, the mice were euthanized, and tumor tissues were collected for weighing and subsequent analysis.

### Annexin V-APC/propidium iodide (PI) staining

SW480, LoVo, and RKO cells were treated with DSF for 48 h and subsequently stained with Annexin V-APC and PI using the Annexin V-APC apoptosis detection kit. The cell concentration was adjusted to 1 × 10^6^ cells/mL, and 1× binding buffer was used throughout the procedure. To each 500 μL of cell suspension, 10 μL of Annexin V-APC and 5 μL of PI or 7-AAD were added. Following incubation in the dark at room temperature for 5 min, flow cytometry analysis was performed immediately.

### TEM

For TEM analysis, SW480, LoVo, and RKO cells were treated with DSF at concentrations of 7 μM, 5 μM, and 13 μM, respectively, for 18 h. The cells were fixed in 4% paraformaldehyde for 1 h, washed three times with PBS buffer (15 min per wash), and subsequently fixed in 1% osmium tetroxide for 2 h. The samples were then dehydrated through a graded ethanol series (50%, 70%, 80%, 90%, and 100% ethanol, each for 10 min), followed by two 10-min incubations in 100% acetone. Next, the samples were infiltrated stepwise with embedding solution: first in a 3:1 mixture of 100% acetone to embedding solution for 0.5 h, then in a 1:1 mixture for 4 h, and finally in pure embedding solution overnight at 4 °C. The samples were embedded in resin at 37 °C for 24 h and polymerized at 60 °C for 48 h. Ultrathin sections approximately 100 nm thick were cut using an ultramicrotome and stained sequentially with uranyl acetate (20 min) and lead citrate (12 min). Imaging data were acquired using a Tecnai G2 Spirit 120 kV transmission electron microscope.

### Immunofluorescence staining

Cells were cultured in confocal dishes, treated with DSF, and subsequently washed twice with PBS. Following this, the cells were fixed with 100 μL of 4% paraformaldehyde for 20 min at room temperature. After fixation, cells were permeabilized with 0.25% Triton X-100 (20 min, RT) after PBS washes. The cells were then washed three times with PBS and blocked with 5% bovine serum albumin (BSA) for 1 h at room temperature to reduce non-specific binding. Next, the cells were incubated overnight at 4 °C with the primary antibody diluted at a ratio of 1:200. Afterward, the cells were washed three times with PBS and incubated with the secondary antibody, diluted at a ratio of 1:2000, for 1 h at room temperature in the dark. Subsequently, the cells were washed three times with PBS, and nuclei were stained with 10 μL of DAPI staining solution per well for 10 min in the dark. Finally, the samples were observed and imaged using a confocal microscope

### RT-qPCR

Total RNA extraction and cDNA synthesis were performed using TRIzol (Thermo Fisher, #15596026) and the Evo M-MLV RT Premix kit (Accurate Biology, AG11706) according to the manufacturer’s instructions. Gene transcription levels were determined using the SYBR Green Pro Taq HS Premix kit (Accurate Biology, AG11701) with the primer sequences (HuaDa Biotechnology). *BECN1* primer sequences are as forward primer 5’-AAGACAGAGCGATGGTAG-3’ and reverse primer 5’-CTGGGCTGTGGTAAGTAA-3’. *FOS* primer sequences are as forward primer 5’-GGAGGGAGCTGACTGATACAC-3’ and reverse primer 5’-AGCTGCCAGG ATGACTCTAG-3’.

### Statistical analysis

All data are presented as mean ± standard deviation (s.d.). Data analysis was performed using the GraphPad Prism 8 software (GraphPad Software, La Jolla, CA, USA). Differences between the control and test groups were analyzed using Student’s *t*-test and one-way analysis of variance (ANOVA), and statistical significance was set at *p* < 0.05.

### Ethics approval and consent to participate

The animal experiments were approved by the Animal Research Ethics Committee of Southern Medical University (IACUC-LAC-20240804-001). All experimental methods were conducted in accordance with relevant guidelines and regulations.

## Supplementary information

Supplementary data

Original WB

Differentially Expressed Genes

## Data Availability

All supporting data are available within the article and supplementary files, or corresponding authors upon reasonable request.
