# Supplementary material for: Disulfiram activates autophagy via proteasome inhibition and c-Fos/beclin-1 upregulation, synergizing with chloroquine
Source: Cell Death Discov. 2025 Dec 12;12:43. doi: 10.1038/s41420-025-02899-7 (PMC12830890; doi:10.1038/s41420-025-02899-7)
Supplement: Supplementary file 1 — Supplementary data [file 41420_2025_2899_MOESM1_ESM.docx]

| 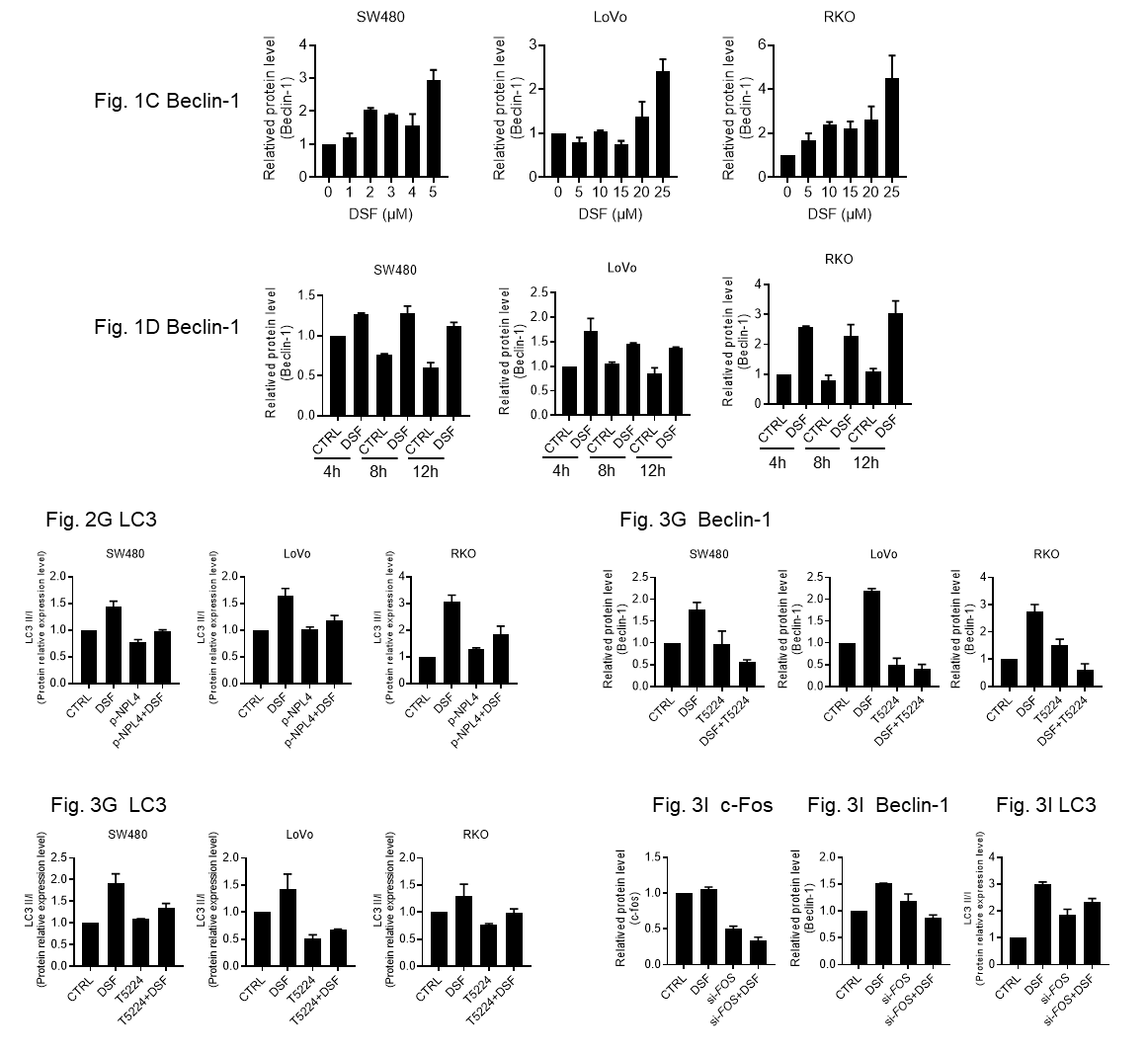 |
| --- |
| **Supplementary Fig. 1** Quantification of the key Western blot results in Fig. 1 - Fig. 3. |

| 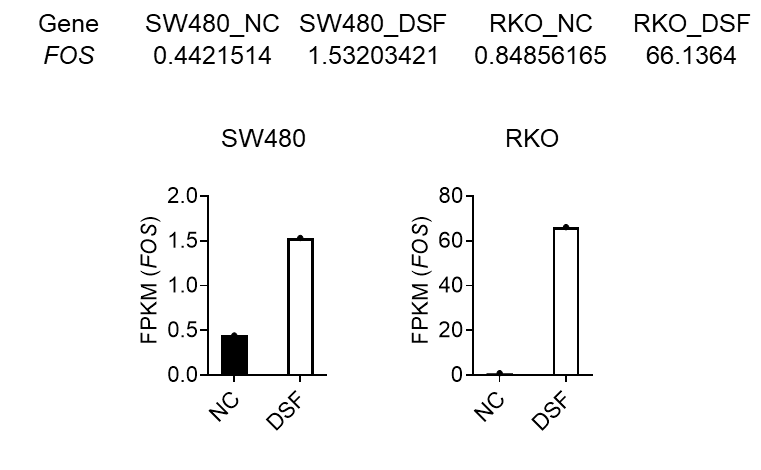 |
| --- |
| **Supplementary Fig. 2** FPKM values of *FOS* in SW480 and RKO cells after DSF treatment. |

| 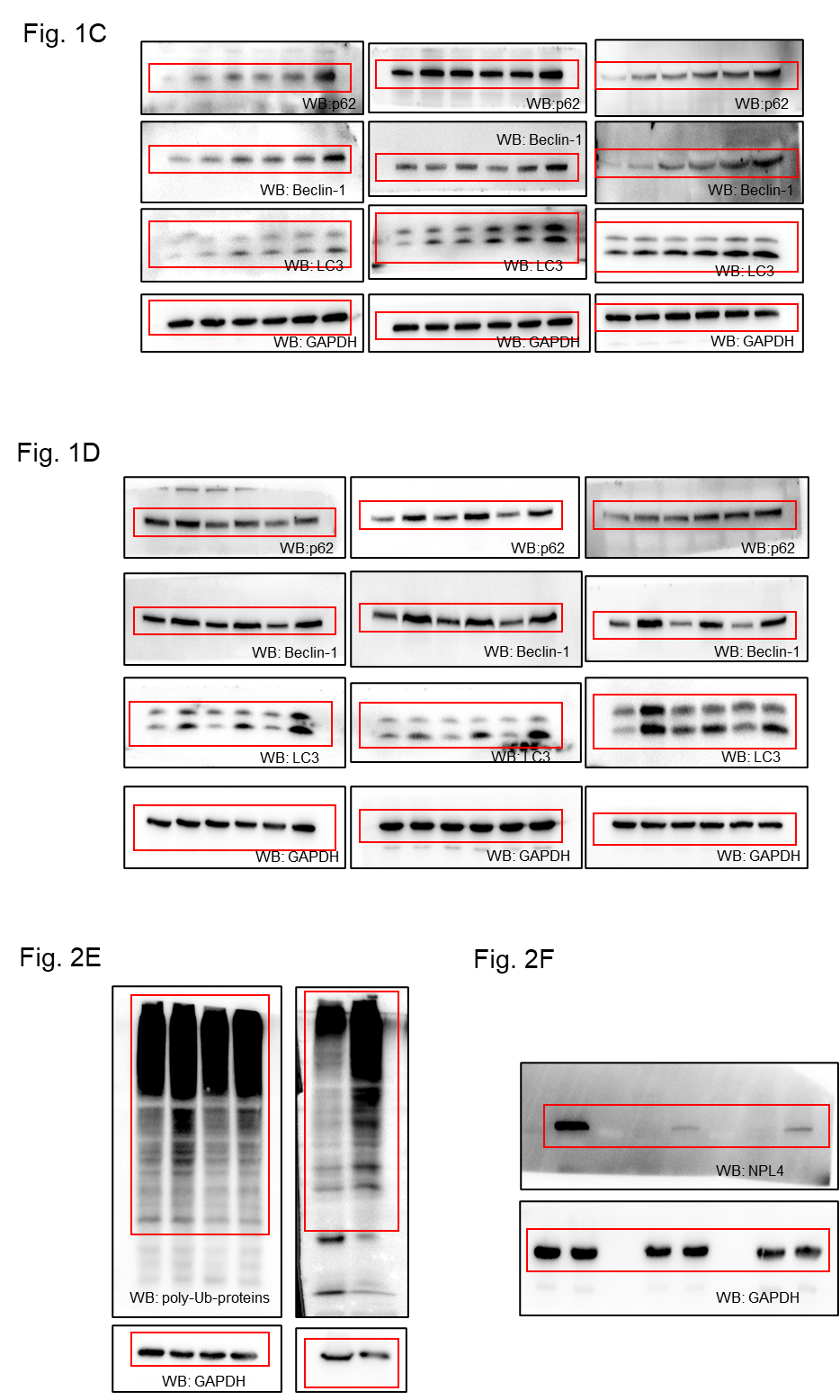 |
| --- |
| **Supplementary Fig. 3 Original Western Blot shown in Figures 1-2.** Each figure corresponds to the Western Blots in the indicated Figure number. |
| 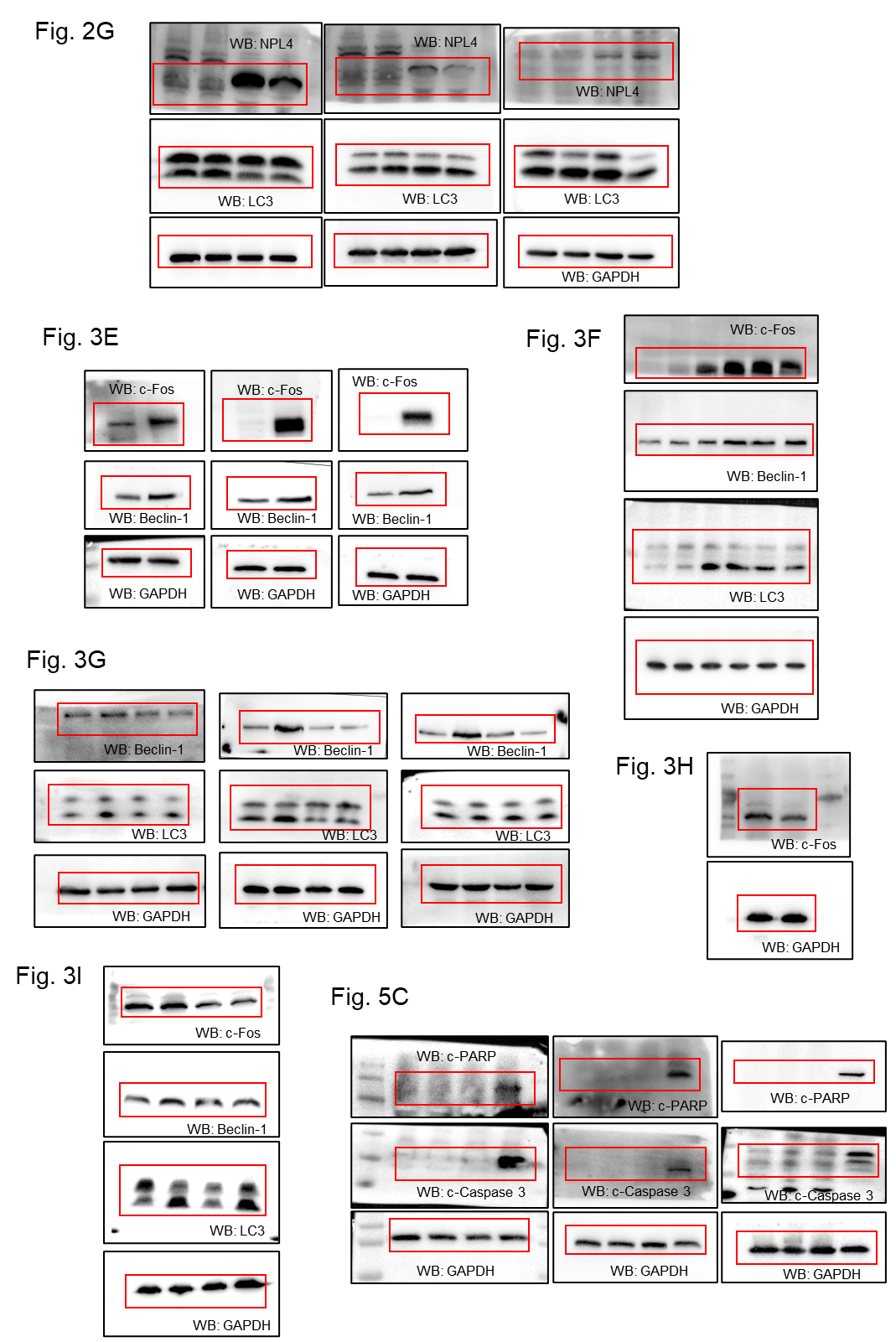 |
| **Supplementary Fig. 4 Original Western Blot shown in Figures 2-5.** Each figure corresponds to the Western Blots in the indicated Figure number. |
